# Supplementary material for: TGFβ links EBV to multisystem inflammatory syndrome in children
Source: Nature. 2025 Mar 12;640(8059):762–71. doi: 10.1038/s41586-025-08697-6 (PMC12003184; doi:10.1038/s41586-025-08697-6)
Supplement: Supplementary file 1 — Supplementary Data 1–18 [file 41586_2025_8697_MOESM1_ESM.zip › 2023-07-11632E-SI Data/2023-07-11632E-SI_Guide_2023-07-11632E-Z.docx]

**SI Guide for Goetzke et al. TGFβ links EBV to Multisystem inflammatory syndrome in children**

**Supporting information 1: Differentially expressed genes of MIS-C patients and 6w p.i. no MIS-C between each cluster at a resolution of 0.6 to define cell clusters.** Given are p-values (p_val, determined by Wilcoxon Rank Sum test), average log2fold change (avg_log2FC), percentage of cells in cluster positive expressing the indicated gene, percentage of all cells positive for this gene (percentage overall), difference of percentages (percentage in cluster – percentage overall), adjusted p value for multiple testing (p_val_adj), cluster number (cluster) and gene name (name).

**Supporting information 2: Differentially expressed genes of cells of patients before and after treatment with methylprednisolone at a resolution of 0.1 to define cell clusters.** Given are p-values (p_val, determined by Wilcoxon Rank Sum test), average log2fold change (avg_log2FC), percentage of cells in cluster positive expressing the indicated gene, percentage of all cells positive for this gene (percentage overall), difference of percentages (percentage in cluster – percentage overall), adjusted p value for multiple testing (p_val_adj), cluster number (cluster) and gene name (name).

**Supporting information 3: Results from Gene set enrichment analysis on a single cell level.** Given are tables for positive enrichment (pos_enrichment) and negative enrichment (neg_enrichment) using a prescreening with an FDR of <0.5 (FDR0.5) or 0.25 (FDR0.25) of samples from MIS-C tested against 6 w. p.i. no MIS-C (and influenza (with_flu)) samples. ID and symbol of gene set, number of (Sign.Cells:_) and percentage of (%Sign.Cells:_) cells with significant gene set enrichment for each cell type (B_cells, T_cells or Monocytes) and group (MIS-C, 6w_pi_no_MIS-C or flu), and the genes that are differentially expressed for each group (CoreGenes:_)are given.

**Supporting information 4: Source data for T-cell reactivity assays.** Given are the source data for Fig. 2 d-f and Extended Data Fig. 7 b+d-e, indicating the peptide pools used in each ARTE-assay.

**Supporting information 5: Source data for HLA-Haplotyping.** Given are the source data for Extended Data Fig. 7 h-j, indicating the exact HLA-haplotypes of patients and controls.

**Supporting information 6: Chi-Squared test results for HLA-Haplotyping.** Given are the p-values for each HLA-A, HLA-B, HLA-C, HLA-DRB, HLA-DQA, HLA-DQB, and HLA-DPB subtype by Fisher’s exact or by Chi-Squared test (MIS-C samples tested against controls).

**Supporting information 7: Characteristics of paediatric controls from Berlin.** Given are the age, gender at birth, SARS-CoV-2 Variant of Concern (VoC), sampling day relative to onset of symptoms (days after symptom), disease severity, result of anti-SARS-CoV-2 antibody testing on day of sampling (Day 1 IgG result), Body-mass-index (BMI in kg/m²), ethnicity, anti-EBV-IgG and -IgM results, and anti-CMV-IgG results.

**Supporting information 8: MIS-C patient characteristics from Berlin cohort.** Given are age in years, sex, BMI in kg/m², BMI z-score, ethnicity, information on which patient within the scRNAseq data the patient corresponds to (included in 10x), time of sampling relative to start of therapy, symptoms, laboratory results and clinical findings, days hospitalized due to MIS-C, highest level of medical care (ICU (intensive care unit), IMC (intermediate care unit) or ward), treatment (IVIG (intravenous immunoglobulins), 1-2 mg/kg or 20-30 mg/kg methylprednisolone, Anakinra, ASA (acetylsalicylic acid), inotropics (including epinephrine, norepinephrine, milrinone)), respiratory support (intubation and ventilation or O_2_ supplementation), result of viremia testing by PCR (EBV, CMV, HHV6, HSV1/2, Parvovirus B19) and serology for EBV, CMV, HHV6, HSV1/2, HHV8, Parvovirus B19, Adenovirus for each patient.

**Supporting information 9: MIS-C patient characteristics from Lyon cohort.** Given are age in years, results of viremia testing by PCR (EBV, CMV, HHV6, HHV8 and Parvovirus B19, and results of serology for EBV, CMV, HHV6, HHV8 and Parvovirus B19 for each patient.

**Supporting information 10: MIS-C patient characteristics from Ankara cohort.** Given are for each patient sex, age in years, ethnicity, BMI in kg/m²m, results from serology testing of EBV and CMV, symptoms (abbreviations: GI (gastrointestinal), CNS (central nervous system)), laboratory results, clinical findings, treatment on a PICU (paediatric intensive care unit), respiratory support and duration of hospitalization due to MIS-C.

**Supporting information 11: MIS-C patient characteristics from Boston cohort.** Given are for each patient: age, sex, ethnicity, symptoms, highest level of care in hospital, cardiac involvement, and results of EBV-serology. (This data was previously published inBartsch, Y.C., Wang, C., Zohar, T. et al. Humoral signatures of protective and pathological SARS-CoV-2 infection in children. Nat Med 27, 454–462 (2021). https://doi.org/10.1038/s41591-021-01263-3 )

**Supporting information 12: Calculations used for age matching of MIS-C patients and controls to account for age-differences in virus seroprevalence.** The top left box (cells A2-C8 (referring to excel sheet “MIS-C vs controls”, but similar on all sheets)) lists the number of patients with MIS-C with positive or negative EBV-serology binned by age-ranges (0-3 years, 3-5 years, 6-14 years, 15-16 years and additionally 17 and older) predefined by the control group. Individual patients can be identified in Extended Data Table 8-12. The top right box (cells K2-M8) and cells M11 to P17 list the children that we analysed from Extended Data Table 8 and from Beer A. 2017 respectively. These two groups are added bin-wise. The result is listed in cells C14 to E17 next to the n numbers for each bin from the MIS-C patients (cells B14-B17) (irrespectively of the result of EBV-serology). In cells F13-H17 the result of the function to match the frequency of patients in each bin for MIS-C and the control group named “healthy” is presented. For cells I14-K17 we rarefied the total patients per bin, so that no patient or control was counted twice, resulting in the final counts listed in I18-K18 which are used for the subsequent statistical analysis.

**Supporting information 13: EBV reads found in the scRNAseq activated B cells/plasmablasts dataset.** Given are the read_ID, the position in the EBV genome, type of read, the EBV gene name that matches the read, and the complete annotation using NC_007605.gff.

**Supporting information 14: EBV reads found in the scRNAseq activated B cells/plasmablasts dataset.** Given is the fasta-file with the sequences identified to match the EBV genome.

**Supporting information 15: MIS-C patient characteristics from Chile and Italy cohort.** Given are for each patient: age, sex and day of sample collection relative to first day of hospitalization.

**Supporting information 16: Lower levels of quantification for 45-Multiplex.** The assay specific lower limits of quantification for the cytokines, chemokines and growth factors detected by the 45-multiplex in pg/ml are provided.

**Supporting information 17: Differentially expressed genes of cells of patients with MIS-C, no-MIS-C controls and patients with acute influenza at a resolution of 0.5 to define cell clusters.** Given are p-values (p_val, determined by Wilcoxon Rank Sum test), average log2fold change (avg_log2FC), percentage of cells in cluster positive expressing the indicated gene, percentage of all cells positive for this gene (percentage overall), difference of percentages (percentage in cluster – percentage overall), adjusted p value for multiple testing (p_val_adj), cluster number (cluster) and gene name (name).

**Supporting information 18: Frequencies and counts of cells per cluster of patients with MIS-C, no-MIS-C controls and patients with acute influenza at a resolution of 0.5.** Given are cell counts and frequencies for each patient and each cluster.
