# Supplementary material for: TGFβ links EBV to multisystem inflammatory syndrome in children
Source: Nature. 2025 Mar 12;640(8059):762–71. doi: 10.1038/s41586-025-08697-6 (PMC12003184; doi:10.1038/s41586-025-08697-6)
Supplement: Supplementary file 2 — Reporting Summary [file 41586_2025_8697_MOESM2_ESM.pdf]

nature portfolio

Corresponding author(s): Carl Christoph Goetzke, Tilmann Kallinich, Mir-Farzin Mashreghi (lead)

Last updated by author(s): Jan 1, 2025

Reporting Summary

Nature Portfolio wishes to improve the reproducibility of the work that we publish. This form provides structure for consistency and transparency in reporting. For further information on Nature Portfolio policies, see our Editorial Policies and the Editorial Policy Checklist.

Statistics

For all statistical analyses, confirm that the following items are present in the figure legend, table legend, main text, or Methods section.

n/a	Confirmed	
☐	☒	The exact sample size (*n*) for each experimental group/condition, given as a discrete number and unit of measurement
☐	☒	A statement on whether measurements were taken from distinct samples or whether the same sample was measured repeatedly
☐	☒	The statistical test(s) used AND whether they are one- or two-sided<br>*Only common tests should be described solely by name; describe more complex techniques in the Methods section.*
☒	☐	A description of all covariates tested
☐	☒	A description of any assumptions or corrections, such as tests of normality and adjustment for multiple comparisons
☐	☒	A full description of the statistical parameters including central tendency (e.g. means) or other basic estimates (e.g. regression coefficient) AND variation (e.g. standard deviation) or associated estimates of uncertainty (e.g. confidence intervals)
☐	☒	For null hypothesis testing, the test statistic (e.g. *F*, *t*, *r*) with confidence intervals, effect sizes, degrees of freedom and *P* value noted<br>*Give P values as exact values whenever suitable.*
☒	☐	For Bayesian analysis, information on the choice of priors and Markov chain Monte Carlo settings
☒	☐	For hierarchical and complex designs, identification of the appropriate level for tests and full reporting of outcomes
☐	☒	Estimates of effect sizes (e.g. Cohen's *d*, Pearson's *r*), indicating how they were calculated

Our web collection on statistics for biologists contains articles on many of the points above.

Software and code

Policy information about availability of computer code

Data collection	Flow cytometry data was aquired using the software of the Multi-Application MA900 Cell Sorter (Sony Biotechnology) and MACSQuantify™ Software of the MACSQuant® Analyzer 16 Flow Cytometer (Miltenyi Biotec). Simple Western Size-Based assays were run on a Wes instrument (proteinsimple; Bio-Techne).
Data analysis	Raw sequence reads were processed using cellranger version 5.0.0. Statistics and data analysis was performed in R (version 4.1.2). Details of used packages are described in the Material and Methods section of the manuscript. Loupe Browser (version 5, 10x Genomics) was used to identify and define cells of interest by manual gating. Flow cytometry data was analysed using FlowJo software 10.8.1 (TreeStar). Simple Western Size-based assays were analysed using the Compass for SW (BioTechne; Protein Simple version 3.1.7).

For manuscripts utilizing custom algorithms or software that are central to the research but not yet described in published literature, software must be made available to editors and reviewers. We strongly encourage code deposition in a community repository (e.g. GitHub). See the Nature Portfolio guidelines for submitting code & software for further information.

Data

Policy information about availability of data

All manuscripts must include a data availability statement. This statement should provide the following information, where applicable:

- Accession codes, unique identifiers, or web links for publicly available datasets
- A description of any restrictions on data availability
- For clinical datasets or third party data, please ensure that the statement adheres to our policy

Next Generation Sequencing data sets are available in the GEO repository GSE254179. . Data was mapped using the human genome reference hg19 [https://www.10xgenomics.com/support/software/cell-ranger/downloads/cr-ref-build-steps]. data sets used for GSEA are available in the Molecular Signatures Database (MSigDB) [https://www.gsea-msigdb.org/gsea/msigdb/] and in the GEO repository under accession number GSE184329. Activated B cell and Plasmablast datasets used from previous studies can be found in the respective repositories: GSE253862 and GSE158038. Source data and additional supporting data are provided with this paper in the supporting information section.

Human research participants

Policy information about studies involving human research participants and Sex and Gender in Research.

Reporting on sex and gender

Participant's sex as self-reported. No sex- and gender-based analyses were performed.

Population characteristics

Pt B01 10 years female
Pt B02 9 years female
Pt B03 9 years male
Pt B04 8 years male
PT B05 10 years female
Pt B06 6 years male
Pt B07 4 years female
Pt B08 3 years female
Pt B09 8 years male
Pt B10 10 years female
Pt B11 4 years male
Pt B12 2 years male
Pt B13 15 years male
Pt B14 5 years male
Pt B15 9 years male
Pt B16 8 years female
Pt B17 7 years female
Pt B18 7 years male
Pt B19 10 years male
Pt B20 8 years male
Pt B21 12 years male
Pt B22 17 years male
Pt B23 10 years male
Pt B24 5 years male
Pt B25 8 years male
Pt B26 7 years male
Pt B27 10 years female
Pt B28 14 years male
Pt B29 1 years male
Pt B30 16 years male
Pt B31 9 years female
Pt B32 19 years male
Pt B33 14 years female
Pt B34 2 years male
Pt B35 2 years female
Pt B36 2 years female
Pt B37 15 years male
Pt B38 18 years male
Pt B39 8 years female

Pt L01	4	years
Pt L02	17	years
Pt L03	10	years
Pt L04	8	years
Pt L05	4	years
Pt L06	11	years
Pt L07	8	years
Pt L08	8	years
Pt L09	7	years
Pt L10	9	years
Pt L11	12	years

Pt L12	9	years		
Pt L13	6	years		
Pt L14	1	years		
Pt L15	10	years		
Pt L16	13	years		
Pt L17	17	years		
Pt L18	11	years		
Pt L19	7	years		
Pt L20	13	years		
Pt L21	3	years		
Pt L22	17	years		
Pt L23	13	years		
Pt L24	5	years		
Pt L25	12	years		
Pt L26	7	years		
Pt L27	10	years		
Pt L28	9	years		
Pt L29	7	years		
Pt L30	12	years		
Pt L31	8	years		
Pt L32	16	years		
Pt L33	8	years		
Pt L34	15	years		
Pt L35	14	years		
Pt L36	9	years		
Pt L37	12	years		
Pt L38	15	years		
Pt L39	6	years		
Pt L40	11	years		
Pt L41	11	years		
Pt L42	9	years		
Pt L43	15	years		
Pt L44	9	years		
Pt L45	5	years		
Pt L46	8	years		
Pt L47	6	years		
Pt L48	9	years		
Pt L49	10	years		
Ankara_01	Male		5	years
Ankara_02	Male		13	years
Ankara_03	Female		15	years
Ankara_04	Male		13	years
Ankara_05	Male		11	years
Ankara_06	Female		16	years
Ankara_07	Male		13	years
Ankara_08	Male		9	years
Ankara_09	Female		8	years
Ankara_10	Male		11	years
Ankara_11	Female		6	years
Ankara_12	Female		7	years
Ankara_13	Male		11	years
Ankara_14	Male		13	years
Ankara_15	Female		11	years
Ankara_16	Female		1	years
Ankara_17	Female		3	years
Ankara_18	Male		6	years
Ankara_19	Female		2	years
Ankara_20	Male		6	years
Boston_1	male		1	years
Boston_2	female		2	years
Boston_3	male		3	years
Boston_4	male		4	years
Boston_5	female		7	years
Boston_6	male		8	years
Boston_7	male		10	years
Boston_8	male		12	years
Boston_9	male		14	years
Chile_01	Male		4	years
Chile_02	Male		8	years
Chile_03	Male		13	years
Chile_04	Male		13	years
Chile_05	Male		4	years
Chile_06	Female		3	years
Chile_07	Male		8	years
Chile_08	Female		6	years
Chile_09	Female		2	years

Chile_10	Male	1	years
Chile_11	Male	12	years
Chile_12	Female	3	years
Chile_13	Female	11	years
Chile_14	Male	2	years
Italy_1	Male	5	years
Italy_2	Male	5	years
COV Pat01	male	10	years
COV Pat02	female	18	years
COV Pat03	female	8	years
COV Pat04	male	15	years
COV Pat05	male	9	years
COV Pat06	male	15	years
COV Pat07	male	8	years
COV Pat08	male	12	years
COV Pat09	female	11	years
COV Pat10	female	7	years
COV Pat11	male	17	years
COV Pat12	male	13	years
COV Pat13	male	12	years
COV Pat14	female	5	years
COV Pat15	male	1	years
COV Pat16	male	12	years
COV Pat17	female	6	years
COV Pat18	female	13	years
COV Pat19	male	11	years
COV Pat20	female	9	years
COV Pat21	female	6	years
COV Pat22	male	6	years
COV Pat23	female	2	years
COV Pat24	male	6	years
COV Pat25	female	10	years
COV Pat26	female	7	years
COV Pat27	male	12	years
COV Pat28	male	5	years
COV Pat29	male	4	years
COV Pat30	male	2	years
COV Pat31	male	1	years
COV Pat32	female	4	years
COV Pat33	male	1	years
COV Pat34	male	5	years
COV Pat35	male	5	years
COV Pat36	female	13	years
COV Pat37	female	6	years
COV Pat38	male	16	years
COV Pat39	male	3	years
COV Pat40	female	8	years
COV Pat41	female	12	years
COV Pat42	female	17	years
COV Pat43	female	6	years
COV Pat44	female	9	years
COV Pat45	female	5	years
COV Pat46	male	4	years
COV Pat47	male	6	years
COV Pat48	female	10	years
COV Pat49	male	11	years
COV Pat50	female	13	years
COV Pat51	female	14	years
COV Pat52	male	1	years
COV Pat53	male	8	years
COV Pat54	female	11	years
COV Pat55	male	10	years
COV Pat56	male	13	years
COV Pat57	male	2	years
COV Pat58	male	1	years
COV Pat59	female	13	years
COV Pat60	female	9	years
COV Pat61	male	2	years
COV Pat62	male	2	years
COV Pat63	male	9	years
COV Pat64	male	16	years
COV Pat65	male	11	years
COV Pat66	male	9	years
COV Pat67	female	8	years
COV Pat68	female	6	years
COV Pat69	male	10	years

COV Pat70	female	10	years
COV Pat71	female	2	years
COV Pat72	female	8	years
COV Pat73	male	11	years
COV Pat74	male	9	years
COV Pat75	female	16	years
COV Pat76	male	6	years
COV Pat77	male	5	years
COV Pat78	male	7	years
COV Pat79	female	11	years
COV Pat80	female	13	years
COV Pat81	male	12	years
COV Pat82	male	10	years
COV Pat83	male	6	years
COV Pat84	female	12	years
COV Pat85	male	10	years
COV Pat86	female	5	years
COV Pat87	female	6	years
COV Pat88	male	12	years
COV Pat89	female	10	years
COV Pat90	male	7	years
COV Pat91	female	15	years
COV Pat92	male	11	years
COV Pat93	female	8	years
COV Pat94	male	11	years
COV Pat95	female	6	years
COV Pat96	female	13	years
COV Pat97	female	2	years
COV Pat98	female	4	years
COV Pat99	female	6	years
HC Pat01	female	7	years
HC Pat02	male	16	years
HC Pat04	male	9	years
HC Pat05	male	9	years
HC Pat06	female	8	years
HC Pat07	female	2	years
HC Pat08	female	10	years
HC Pat09	female	6	years
HC Pat10	male	9	years
HC Pat11	female	9	years
HC Pat12	male	7	years
HC Pat13	female	5	years
HC Pat14	female	7	years
HC Pat15	female	3	years
HC Pat16	male	10	years
HC Pat17	female	10	years
HC Pat18	male	6	years
HC Pat19	female	6	years
HC Pat20	female	15	years
HC Pat21	female	15	years
HC Pat22	female	5	years
HC Pat23	male	10	years
HC Pat24	male	6	years
HC Pat25	male	14	years
HC Pat26	female	8	years
HC Pat27	female	3	years
HC Pat28	male	15	years
HC Pat29	male	9	years
HC Pat30	female	9	years
HC Pat30	male	9	years
HC Pat31	female	4	years
HC Pat32	male	9	years
HC Pat33	female	3	years
HC Pat34	female	6	years
HC Pat35	female	17	years

Recruitment

All patients and/or their legal guardians treated for MIS-C at the Charité Universitätsmedizin Berlin Department of Pediatric Respiratory Medicine, Immunology and Critical Care Medicine were asked by the treating physicians if they would take part in this study. All that agreed were included in the study. Similarily, 49 patients were recruited in Lyon and 20 patients were recruited in Ankara, Turkey, 2 patients in Turin, Italy and 114 patients in Santiago, Chile. Blood samples were taken from the acute phase of MIS-C and during follow-up visits in the out-patient clinic. Additionally, samples from 36 healthy children, 57 children with asymptomatic or mild SARS-CoV-2 infection and 39 children with a moderate and 2 with a severe infection with SARS-CoV-2 during acute and follow up and 11 children 6 weeks post infection with SARS-CoV-2 were used as controls. The

controls were recruited in Berlin in outpatient practices and in paediatric departments.

Ethics oversight	This study was approved by the local institutional review boards (IRB) of: the Charité (Pa-COVID-19 and EA2/178/22) (Berlin, Germany), the Comité de Protection des Personnes Sud Méditerranée I, Marseille, France) (ID-RCB: 2020-A01102-37) for the French patients and by the ethical committee of Hospices civils de Lyon (Lyon University Hospitals, France) N° 23_5231 for French controls, by the Hacettepe University Ethical Committee (2021/09-45, Ankara, Turkey) for MIS-C patients from Turkey, by Mass General Brigham IRB 2020P000955 for the patients and controls from Boston, USA and by the Ethics Committee Federico II in Naples as a collaborative study protocol with the NIH in 2020 (158/2020); and by the ethical committee of Clínica Alemana Universidad del Desarrollo (IRB ID: 202098) for the samples from Chile. Written and informed consent was provided by all legal representatives of the patients that participated in this study.

Note that full information on the approval of the study protocol must also be provided in the manuscript.

Field-specific reporting

Please select the one below that is the best fit for your research. If you are not sure, read the appropriate sections before making your selection.

☒ Life sciences ☐ Behavioural & social sciences ☐ Ecological, evolutionary & environmental sciences

For a reference copy of the document with all sections, see nature.com/documents/nr-reporting-summary-flat.pdf

Life sciences study design

All studies must disclose on these points even when the disclosure is negative.

Sample size	Blood sample size was determined by weight and age of patients included in this study according to "ETHICAL CONSIDERATIONS FOR CLINICAL TRIALS ON MEDICINAL PRODUCTS CONDUCTED WITH THE PAEDIATRIC POPULATION". Number of study participants was determined by the number of patients hospitalised for MIS-C who volunteered to take part in this study.
Data exclusions	Single-cell sequencing data from one patient was excluded as this patient was diagnosed with Kawasaki-Disease instead of MIS-C. For allocating EBV-positive cells to patients, samples where all viral RNA detected was within lytic cells or less than 50 intact total cells per patient were sequenced, were excluded for testing frequencies of positivity, but were included in the overall analysis.
Replication	We report consistent results from the analysis of different subjects. For TGF-β-activity assay two different T-cell donors and 4 different MIS-C patient sera were used. For scRNAseq data 11 MIS-C patients and 4 controls and 3 patients with influenza were used. For T-cell reactivation assay PBMC from 6 different healthy donors with 6 different peptides and treated with serum from 7 different MIS-C patients, or during acute phase and follow up 8 patients with MIS-C with 5 different peptides, or 6 children infected with SARS-CoV-2 with 6 different peptides during the acute phase and during follow-up. For testing of TCRVb21.3 T cells among activated T cells samples from 32 patients (13 Chile, 19 Europe) were used. For the TCR-atlas 5 donors and 2 different peptides and 2 sets of 3 donors with 1 peptide each were used. For EBNA2-peptide reactivation assay 7 different donors in two independet expriments were used. For killing assay of LCL by T cells 4 donors each analyzed twice with blood drawn at different time points were used. For age matched comparison of seroprevalence Age-matched group sizes: MIS-C=62, 64, or 27; control=2573; no MIS-C=75; pCOVID-19 high TGF-β=6 patients or controls were included. For activated B cell/plasmablast datasets (6 healthy/mild COVID-19, 7 Tdap, 15 Comirnaty, 8 Vaxzevria, 32 MIS-C, 12 severe COVID-19 donors were used.
Randomization	Serum samples and PBMCs from patients with MIS-C used in this study were randomly used after a preselection of the samples with high enough cell counts or high enough quantities of serum needed for the experiments.
Blinding	Blinding was not possible in this study due to the inherent nature of the research and the sample processing workflow. Specifically, the study involved analyzing samples from patients with MIS-C and control individuals, obtained from differnt hospitals, outpatient practices and biobanks, each using different tubes and pseudonyms. To minimize potential bias, the researchers performing bioinformatics analysis or analysis of in vitro studies did not have access to patient information during the experimental procedures. Clinical history and additional patient data were only integrated into the dataset after the analysis to contextualize the results.

Reporting for specific materials, systems and methods

We require information from authors about some types of materials, experimental systems and methods used in many studies. Here, indicate whether each material, system or method listed is relevant to your study. If you are not sure if a list item applies to your research, read the appropriate section before selecting a response.

Materials & experimental systems

n/a	Involved in the study
☐	☒ Antibodies
☐	☒ Eukaryotic cell lines
☒	☐ Palaeontology and archaeology
☒	☐ Animals and other organisms
☒	☐ Clinical data
☒	☐ Dual use research of concern

Methods

n/a	Involved in the study
☒	☐ ChIP-seq
☐	☒ Flow cytometry
☒	☐ MRI-based neuroimaging

Antibodies

Antibodies used

Flow cytometry anti-human antibodies:
FITC anti-CD3 (1:100, UCHT1, in house), PerCP-Cy5.5 anti-CD14 (1:50, TÜK4, BioLgend), APC anti-CD38 (1:25, HIT2, Biolegend), APC-Vio770 anti-HLA-DR (1:100, AC122, Miltenyi Biotec), V500 anti-CD19 (1:200, HIB19, BD Biosciences), PE anti-CD27 (1:100, M-T271, BioLegend), CD14 VioBlue (1:200, REA599, Milenyti Biotec), CD19 VioBlue (1:100, REA675, Miltenyi Biotec), CD45Ro BV510 (1:50, UCHL1, BD Biosciences), CD3 PE Cy5 (1:200, UCHT1, BioLegend), CD8a BV650 (1:100, RPA-T8, BioLegend), CD4 PerCPeFlour710 (1:200, SK3, eBioscience), CD69 APC-Cy7 (1:100, FN50, BioLegend), CD154 PE (1:100, REA238, Miltenyi Biotec) CD137 PE Cy7 (1:100, 4B4-1, BioLegend), PE-anti Biotin (1:100, Bio3-18E7, Miltenyi Biotec), CD154 PE (1:40, REA238, Miltenyi Biotec), CD14 VioBlue (1:200, REA599, Milenyti Biotec), CD19 VioBlue (1:100, REA675, Miltenyi Biotec), CD3 PE Cy5 (1:200, UCHT1, BioLegend), CD69 APC (1:100, REA824, Miltenyi Biotec),CD8a BV650 (1:100, RPA-T8, BioLegend), CD4 PerCPeFlour710 (1:200, SK3, eBioscience), TCR Vβ21.3 APCVio770 (1:100, REA894, Miltenyi Biotec), CD107a AlexaFlour647 (1:3000, H4A3, BioLegend), CD3 PE Cy5 (1:200, UCHT1, BioLegend), CD8a BV650 (1:100, RPA-T8, BioLegend), CD4 PerCPeFlour710 (1:200, SK3, eBioscience). active caspase-3 AlexaFlour647 (1:50, BD Bioscience), HLA-DR PerCP-Cy5.5 (1:50, clone L243, BioLegend), CD21 PE-Vio770 (1:75, clone HB5, Miltenyi Biotec), or CD14 AlexaFluor700 (1:500, clone TM1, in house).

CITE-Seq anti-human antibodies:
Hashtag 1, clone LNH-94; 2M2, GTCAACTCTTTAGCG, BioLegend, Cat. 394661; Hashtag 2, clone LNH-94; 2M2, TGATGGCCTATTGGG, BioLegend, Cat. 394663; Hashtag 3, clone LNH-94; 2M2, TTCCGCCTCTCTTTG, BioLegend, Cat. 394665; Hashtag 4, clone LNH-94; 2M2, AGTAAGTTCAGCGTA, BioLegend, Cat. 394667; Hashtag 5, clone LNH-94; 2M2, AAGTATCGTTTCGCA, BioLegend, Cat. 394669; Hashtag 6, clone LNH-94; 2M2, GGTTGCCAGATGTCA, BioLegend, Cat. 394671; Hashtag 7, clone LNH-94; 2M2, TGTCTTTCCTGCCAG, BioLegend, Cat. 394673; Hashtag 8, clone LNH-94; 2M2, CTCCTCTGCAATTAC, BioLegend, Cat. 394675; Hashtag 9, clone LNH-94; 2M2, CAGTAGTCACGGTCA, BioLegend, Cat. 394677; Hashtag 10, clone LNH-94; 2M2, ATTGACCCGCGTTAG, BioLegend, Cat. 394679. All used accoding to manufacturer's reccommended dilutions.

ELISA detection anti-human antibodies:
IgG: HSV1 (Abnova, KA0229), HSV2 (Abnova, KA0231), EBNA1 (abcam, ab108731), CMV (Abnova, KA1452), HHV6 (Abnova, KA1457), Adenovirus (Creative Diagnostics, DEIA2382). All working stock sultions provided and used undiluted.
IgM: HSV1/2 (Abnova, KA4842), EBNA1 (Abnova, KA1449), CMV (Abnova, KA0228), HHV-6 (Creative Diagnostics, DEIABL57), Adenovirus (Creative Diagnostics, DEIA1767) All working stock sultions provided and used undiluted.

In hospital data for virus serology was determined by accredited Immunoblotting Assays for EBV-IgM and –IgG antibodies and enzyme immunoassays by automatic analysis (Liaison, Diasorin and Architect, Abbott).

Neutralising Antibody:
TGF-β1, TGF-β2 and TGF-β3 (50µg/ml, R&D Systems, MAB1835-SP)

antibodies for polyclonal stimulation of T-cells:
Dynabeads™ human T-Activator CD3/CD28 (ThermoFisher)

antibodies for ARTE Assay:
CD40 Antibody, anti-human, pure-functional grade,130-094-133, Miltenyi Biotec
CD28 Antibody, anti-human, pure-functional grade,130-093-375, Miltenyi Biotec

antibodies for size-based protein assays:
SMAD2/3 (clone: D7G7) XP® Rabbit mAb #8685 and Phospho-SMAD2 (Ser465/467)/SMAD3 (Ser423/425) (clone: D27F4) Rabbit mAb #8828 (both 1:100; both Cell Signaling)
anti-β-Tubulin (polyclonal antibody: NB600-936; 1:100; Novus Biologicals)
Anti-Rabbit Secondary HRP Antibody (042-206; Bio-Techne; provided as working stock dilution from manufacturer)

Validation

All purchased antibodies were validated by their manufacturers and further in-house testing for flow cytometry antibodies was done. Titration tests using healthy donors were performed prior to experiments to find optimal working dilutions.

Miltenyi Biotec
"All our antibodies are rigorously tested and validated before release. In the application section on the product page, you can find examples of typical performance data. In addition, we provide extended validation data highlighting details of antibody performance, specificity, and fixation compatibility. All antibodies for which any of these datasets are already available will be indicated with the extended validation stamp."
Validation of antibody specificity by:
- Counterstaining

- Knockout of target protein
- Epitope competition assay
- siRNA knockdown
- Stimulation of cells
- Overexpression of target protein
- Binding to purified antigen (latex bead coating)
- Cross-reactivity

BioLegend Flow Cytometry
"Specificity testing of 1-3 target cell types with either single- or multi-color analysis (including positive and negative cell types). Once specificity is confirmed, each new lot must perform with similar intensity to the in-date reference lot. Brightness (MFI) is evaluated from both positive and negative populations.
Each lot product is validated by QC testing with a series of titration dilutions."
BioLegend TotalSeq™ Antibodies
"Bulk lots are tested by PCR and sequencing to confirm the oligonucleotide barcodes. They are also tested by flow cytometry to ensure the antibodies recognize the proper cell populations.
Bottled lots are tested by PCR and sequencing to confirm the oligonucleotide barcodes."

BD Biosciences
"The specificity is confirmed by using multiple applications that may include a combination of flow cytometry, immunofluorescence, immunohistochemistry or western blot to test a combination of primary cells, cell lines or transfectant models."
"Once our research and development (R&D) team completes evaluation of a new product, the developed process is transferred to our manufacturing teams, including Quality Control."
"Quality control testing of new, manufactured lots are performed side-by-side with a previously accepted lot as a control, helping to serve as a reference for comparison and assuring that performance of the new lot is both reliable and consistent."
"Our strict adherence to these guidelines helps ensure that different lots of conjugated reagents are performing consistently."

Cell Signaling Technology antibodies:
"Antibody Validation for Western Blotting
Western blotting remains one of the most common scientific methods for monitoring protein expression in cells or tissue. The accuracy of western blot results relies heavily of the quality of the primary antibody employed in the immunoblotting. Cell Signaling Technology (CST) provides the highest quality primary and secondary antibodies available for western blotting. CST™ antibodies are produced in-house and validated extensively according to a rigorous protocol.

Validation Steps Include
Examination of several cell lines and/or tissues of known expression levels allows accurate determination of species cross-reactivity and verifies specificity.
Treatment of cell lines with growth factors, chemical activators or inhibitors, which induce or inhibit target expression, verifies specificity. Phosphatase treatment confirms phospho-specificity.
The use of siRNA transfection or knockout cell lines verifies target specificity.
Side-by-side comparison of lots to ensures lot-to-lot consistency.
Optimal dilutions and buffers are predetermined, positive and negative cell extracts are specified, and detailed protocols are already optimized, saving valuable time and reagents."

Novus Biologicals Antibodies:
"Antibody Reproducibility Initiative
Novus recognizes the need for highly validated, high quality antibodies in the life sciences community. The research community faces ongoing concerns about data reproducibility and especially the validity of antibody-based assays. A recent article in Nature discusses the variable standards and performance of antibodies and antibody suppliers in the market. Novus is committed to addressing this problem and to helping our customers attain the best possible results with our products.

To that end, we actively seek high quality, highly validated products and provide support to ensure that our customers have the tools to properly validate their own assays. We are also collaborating with several global initiatives that help life science researchers choose antibodies with proven results. Of the five pillars of validation established by these initiatives, genetic knockout validation provides the most reliable control for assessing antibody specificity."

Antibodies for Size-based Protein assay were checked for external validation using the Simple Western Antibody Database. (https://www.bio-techne.com/resources/simple-western-antibody-database).

Abcam EBNA1 ELISA:
"All kit components have been formulated and quality control tested to function successfully as a kit."

Abnova ELSIA kits:
"The manufacturer guarantees the applicability of the kit as a whole. "

Creative Diagnostics ELISA kits:
"Creative Diagnostics is an evolving biotech company providing highly purified protein/ recombinant antigens worldwide. The protein/ recombinant antigens are rigorously tested to meet the research and development demand for excellent quality, uncompromising biological activity at competitive prices."

Eukaryotic cell lines

Policy information about cell lines and Sex and Gender in Research

Cell line source(s)	Lymphoblastic cell line derived from healthy donors included in this study.<br>And HEK293T cells originally obtained from ATCC (CRL-3216 ™)
Authentication	By Flowcytometry
Mycoplasma contamination	Cells were not tested for mycoplasma contamination during the time of the study.
Commonly misidentified lines<br>(See ICLAC register)	no commonly misidentified cell lines were used in this study.

Flow Cytometry

Plots

Confirm that:

☒ The axis labels state the marker and fluorochrome used (e.g. CD4-FITC).

☒ The axis scales are clearly visible. Include numbers along axes only for bottom left plot of group (a 'group' is an analysis of identical markers).

☒ All plots are contour plots with outliers or pseudocolor plots.

☒ A numerical value for number of cells or percentage (with statistics) is provided.

Methodology

Sample preparation	Pereipheral blood samples for cell sorting:<br>PBMC were isolated from peripheral blood by Ficoll-Paque™ PLUS (Cytiva) density gradient centrifugation at room temperature. Cells were either used directly for analysis or stored at −80°C in heat-inactivated foetal bovine serum (FCS; Corning, 35-079-CV) with 10% v/v dimethylsulfoxide before analysis. Serum samples were stored at −80°C before analysis. 2µg/mL actinomycin D was added to the buffer used during the first centrifugation. Enriched cells were incubated with Fc Blocking Reagent (Miltenyi Biotec) following manufacturer's instructions and subsequently stained for 30 min at 4°C with fluorophore-coupled anti-human antibodies and/or fluorophore-coupled proteins. To stop the staining, cells were washed with PBS/1%BSA. DAPI was added before sorting to allow dead cell exclusion.
Instrument	Sortings were preformed using a MA900 Multi-Application Cell Sorter (Sony Biotechnology), a BD AriaII (BD Biosciences) or a Cytek Aurora (Cytek Biosciences). Cell counting was performed using a MACSQuant16 flow cytometer (Miltenyi Biotec). Flow cytometry analysis was performed using a MACSQuant16 flow cytometer (Miltenyi Biotec).
Software	Flow cytometry data was aquired using the software of a Multi-Application MA900 Cell Sorter (Sony Biotechnology) and MACSQuantify™ Software of the MACSQuant® Analyzer 16 Flow Cytometer (Miltenyi Biotec), or<br>BD FACSDiva™ Software on a BD ARIA II (BD Biosciences) or SpectroFlo® on a Cytek Aurora (Cytek Biosciences) machine, and analysed using FlowJo software 10.8.1 (TreeStar).
Cell population abundance	Cell population abundance was highly variable among subjects. Sorted population purity was analyzed during post-sorting cell counting using a MACSQuant flow cytometer (Miltenyi Biotec).
Gating strategy	For sequencing:<br>cells were identified by FSC-SSC gating and doublets were excluded based on SSC-A vs SSC-H or FSC-A vs FSC-H plots.<br>Activated T-cells were identified as DAPI-, CD14-, CD19-, CD3+, CD38+ and HLA-DRhigh,<br>memory B-cells were identified as DAPI-, CD14-, CD3-, CD19+, CD27+ (and CD38high for plasmablasts),<br>monocytes were identified as DAPI-, CD14+,<br>lineage- HLA-DRhigh cells were identified as DAPI-, CD14-, CD19-, CD3-, and HLA-DRhigh<br>B cells were identified as DAPI-, CD14-, CD3-, CD19+ and CD21+<br>antigen-specific T-cells:<br>Cells were identified by size and granularity in a FSC-vs SSC plot, followed by doublet exclusion in an FSC-A vs. FSC-H plot. Dump (DAPI, CD14 and CD19)+ cells were also excluded. As CD3 is downregulated after T-cell activation (SEB plot in second row), the gate was extended to include CD3low CD45Ro+ cells. CD4+ epitope-specific T-cells were identified as CD69+ CD154 + and CD8+ epitope specific T-cells were identified as CD69+ CD137+. SEB was used as a positive control for correct gating.<br><br>T-cells for co-culture with LCL:<br>Viable (DAPI-) and VioBlue-, CD3+ cells were further sorted according to CD4, CD8 and TCR Vβ21.3 expression<br><br>Viable LCL were defined as CFSE+, fixable viability dye- and active caspase-3- cells

☒ Tick this box to confirm that a figure exemplifying the gating strategy is provided in the Supplementary Information.
